# Hot Water Injections in Uterine Diseases

**Published:** 1875-01

**Authors:** 


### HOT WATER INJECTIONS IN UTERINE DISEASES.

Dr. T. A. Emmet (New York *Medical Journal*), in an interesting and valuable article upon the Philosophy of Uterine Diseases, makes the following practical remarks upon hot water as a means of controlling pelvic circulation and imparting tone to the pelvic vessels :

The *prolonged* use of hot water is followed by a tonic contraction of the arterioles, and thus an approach to healthy action. The immediate effect of heat is dilatation, the secondary effect contraction. The best method of using hot water to obtain its contractile effect the Doctor describes as follows :

The woman is placed on her back, with the hips elevated by a properly shaped bed-pan under her, and a gallon or more of hot water at 98° or a higher temperature, is slowly injected into the vagina by means of Davidson's syringe. This operation blanches the mucous membrane and diminishes the size of the canal, as if strong astringents had been used. While the hips are elevated, the vagina will retain during the injection a large quantity of water, which, by its weight, will distend every portion of the canal, so that it will come in direct contact with the mucous membrane, under which the capillaries lie. The vessels of the neck and body of the uterus pass along the sulcus on each side of the vagina, and their brances encircle the canal in a most complex net-work. The vessels of the fundus, through the veins of which the blood passes by the liver back into the general circulation, communicate with those below by anastomosis.

We can thus, through the vagina, influence, directly or indirectly, the whole pelvic circulation. We can so diminish the supply as not only to check congestion, but we can literally starve out an inflammation. I know from my own personal observation that several of these injections a day at 100° to 106° will abort an attack of cellulitis, if resorted to early enough and their use persevered in, with the use of rest and anodynes. These injections exercise a most beneficial effect on the reflex system, by allaying local irritation. I know no better means for removing the nervousness and sleeplessness of an hysterical woman than a prolonged hot water vaginal injection when administered by an experienced hand. The injections will frequently soothe a patient in less time than could be done by any drug in the pharmacopœia. To receive permanent benefit from their use, they must be continued until the patient is restored to health. They should be given once a day, preferable at bed-time. The only position in which the patient can receive any benefit from them is on the back, with the hips elevated, as described. She cannot administer them properly herself—and I know of no arrangement which can take the place of an intelligent nurse. As the patient improves in health, the quantity of water can be diminished and the temperature lowered, until the injections are discontinued from daily use, but for some time they should be employed for a few days after each period.—*Detroit Med. Review.*

---

PEDICULI.—A solution of oxide of mercury in oleic acid, by its great penetrating power, destroys at once pediculi and ova—a result not always certain when ointments are used.—*Braithwaite's Retrospect.*

